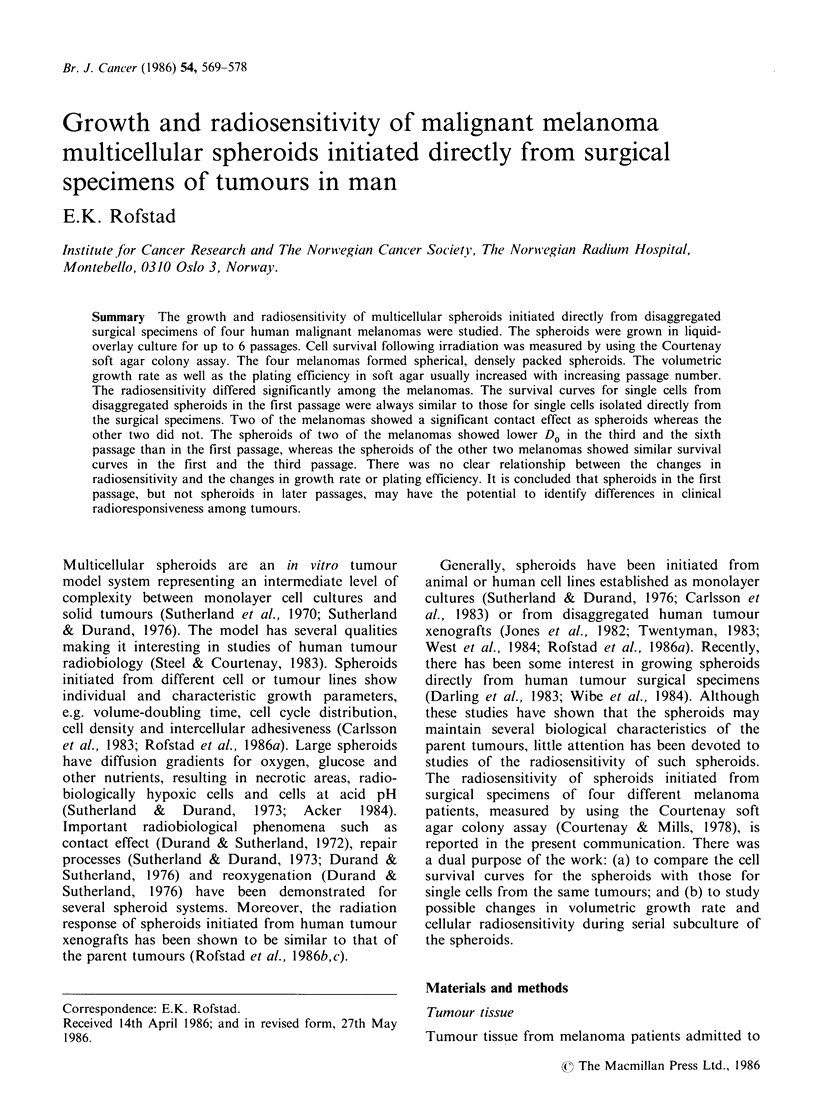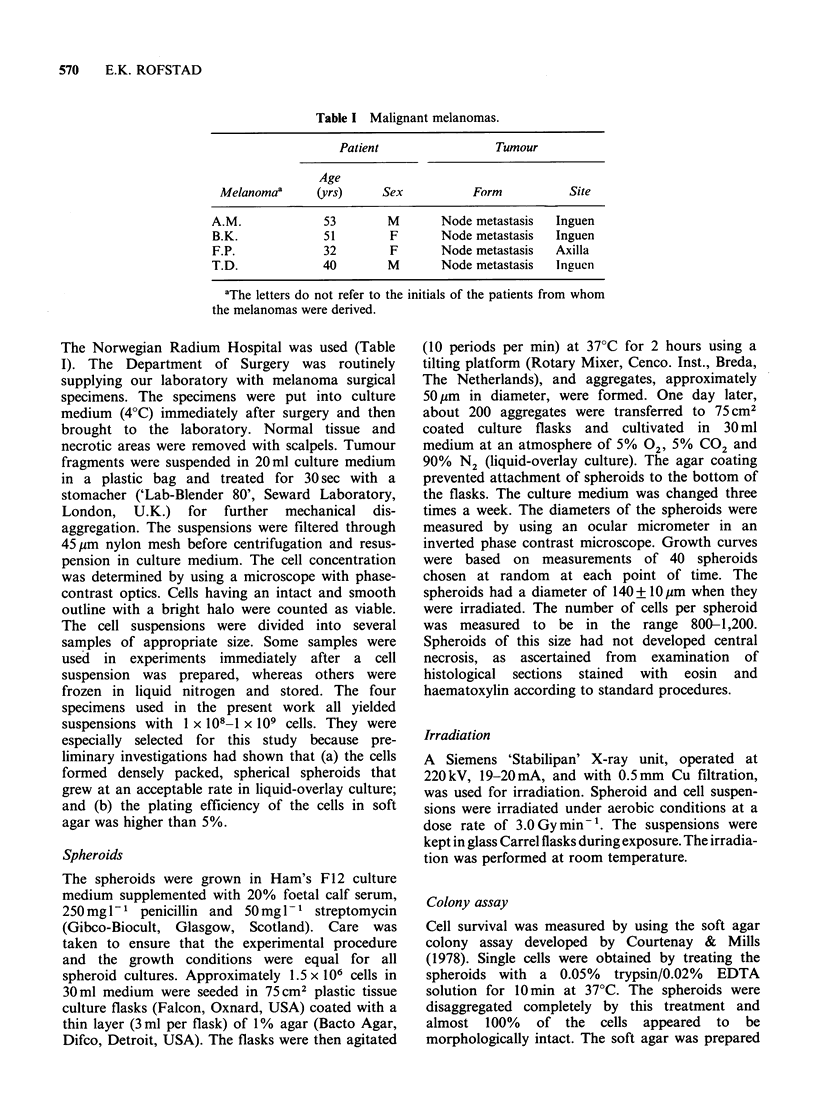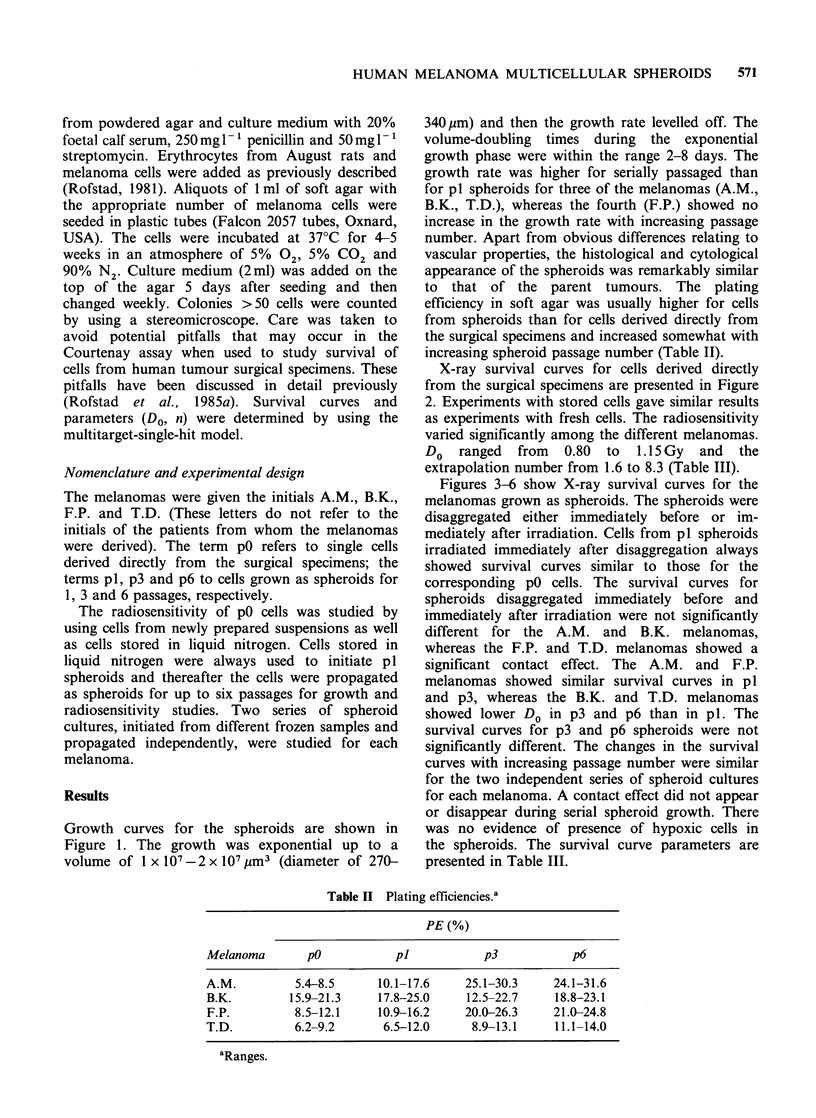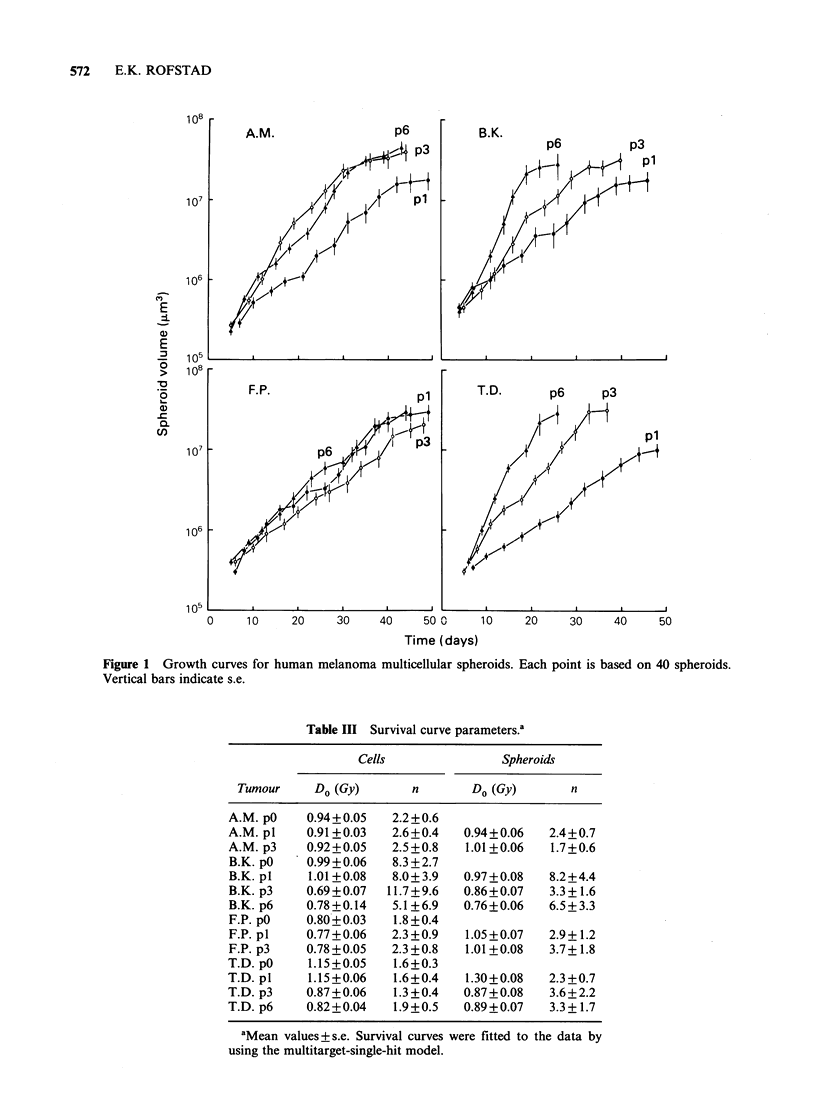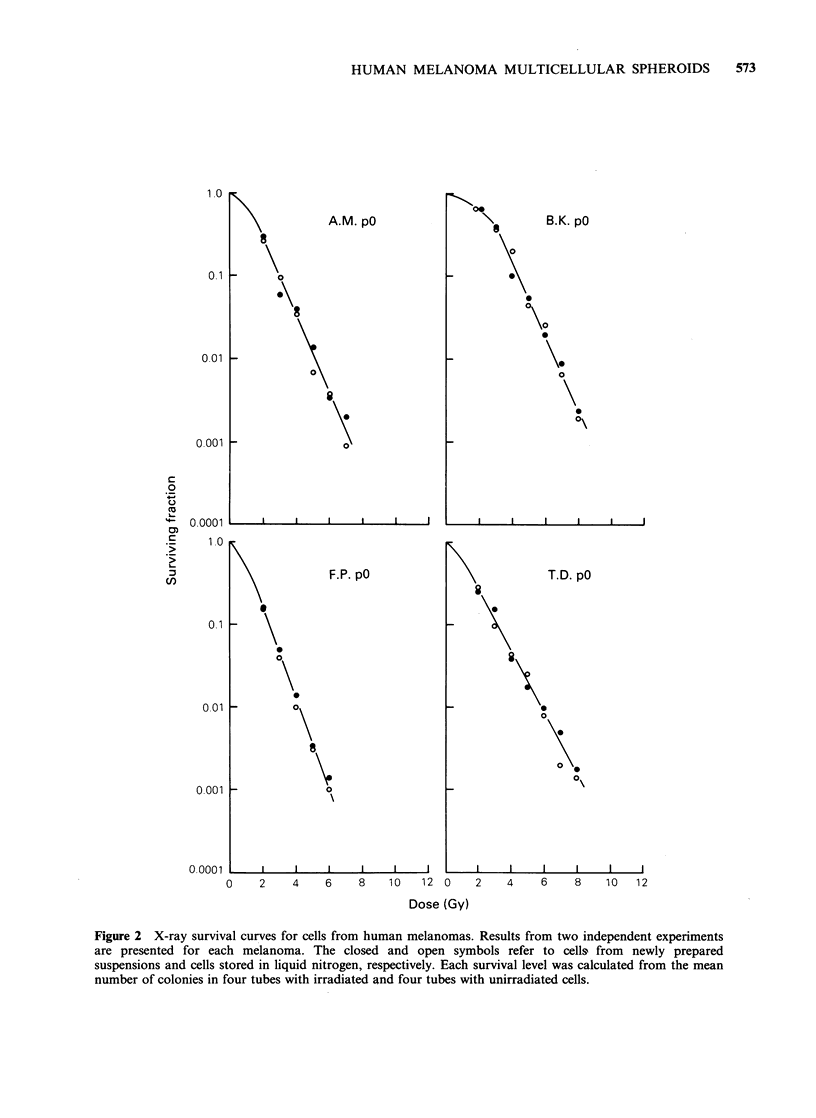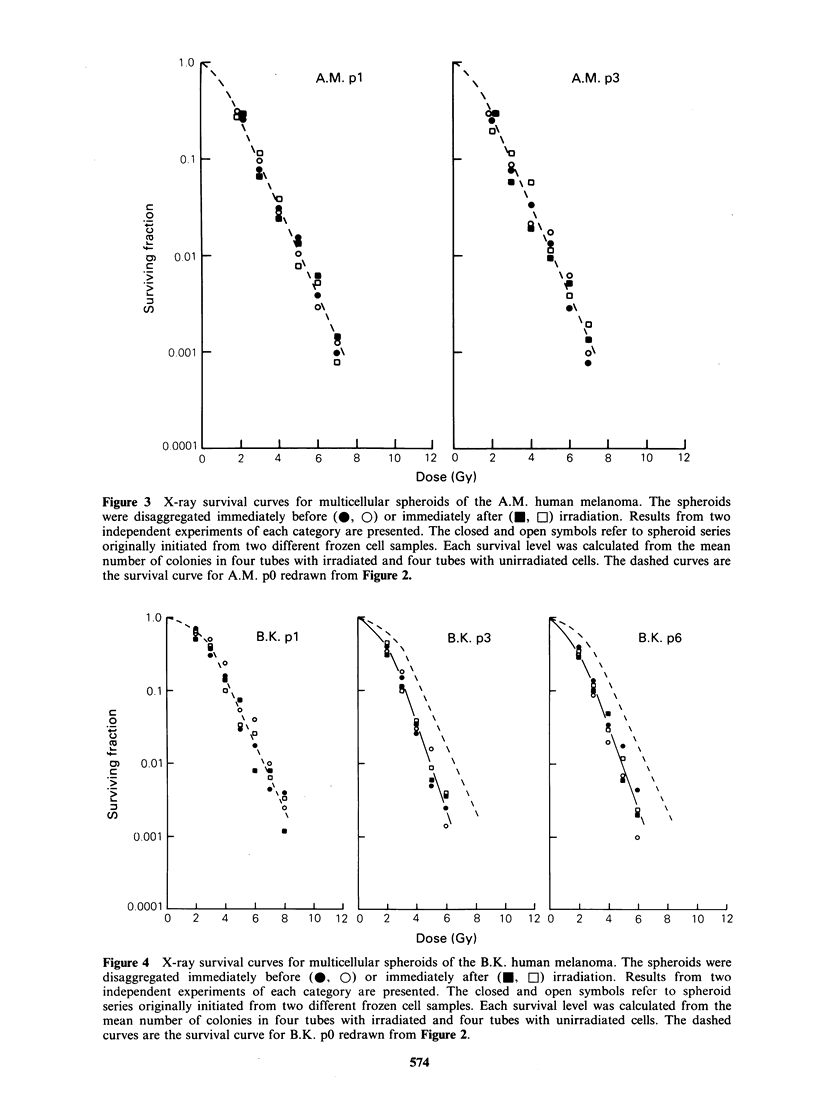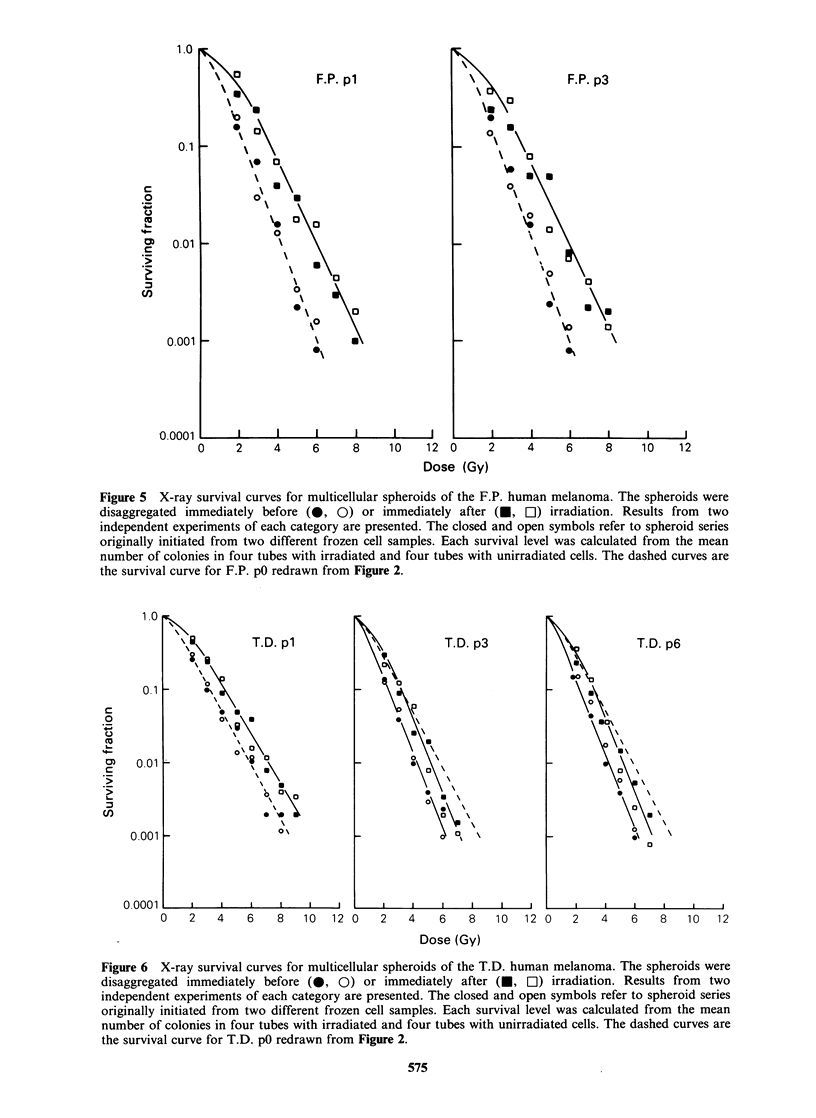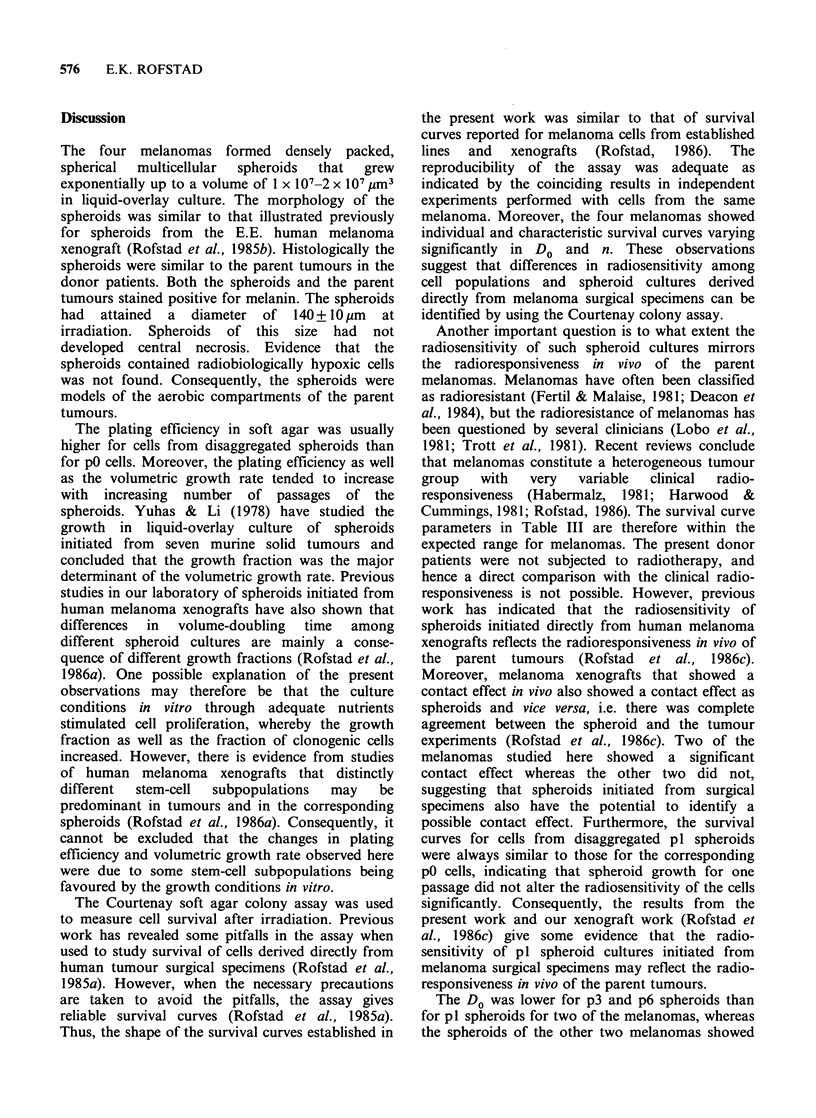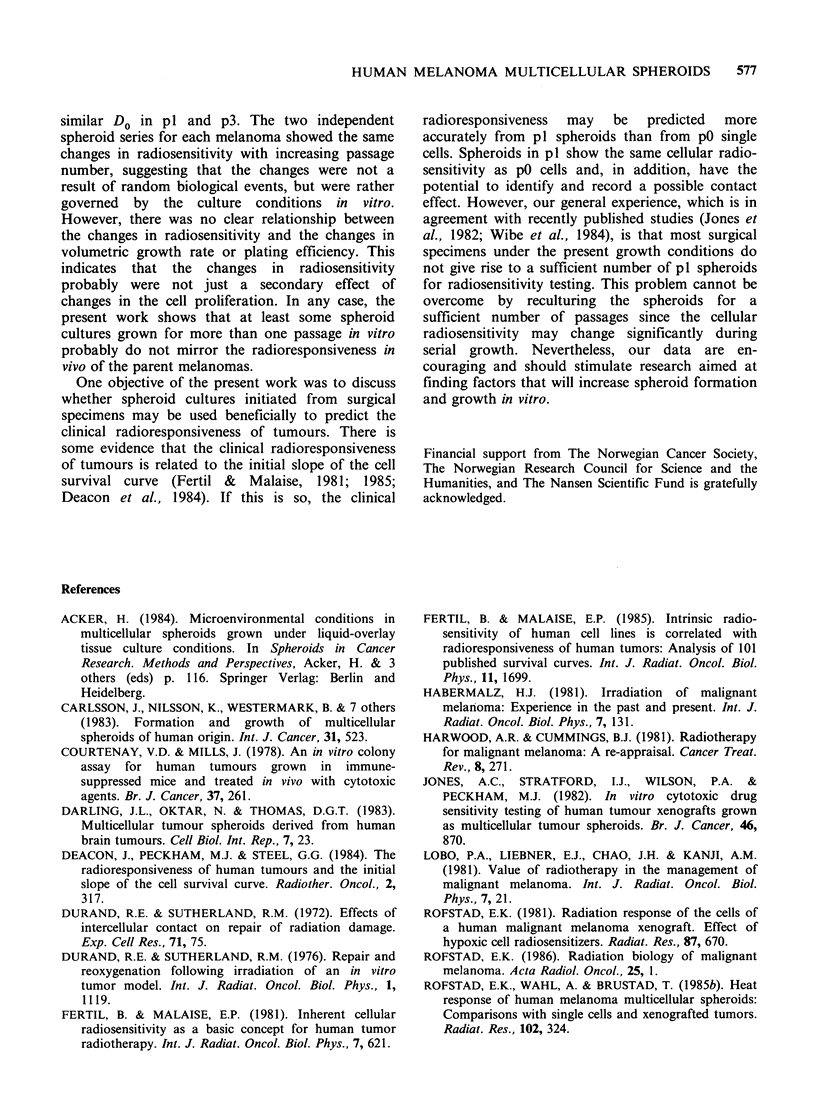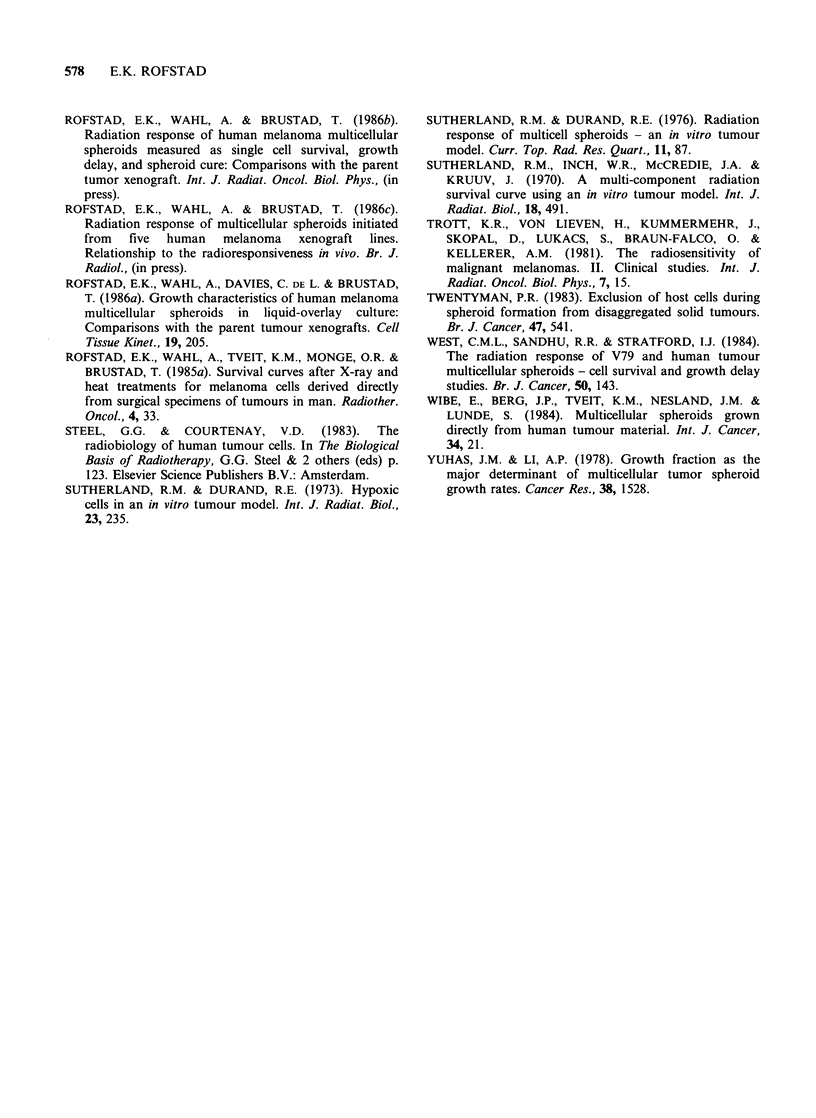# Growth and radiosensitivity of malignant melanoma multicellular spheroids initiated directly from surgical specimens of tumours in man.

**DOI:** 10.1038/bjc.1986.211

**Published:** 1986-10

**Authors:** E. K. Rofstad

## Abstract

The growth and radiosensitivity of multicellular spheroids initiated directly from disaggregated surgical specimens of four human malignant melanomas were studied. The spheroids were grown in liquid-overlay culture for up to 6 passages. Cell survival following irradiation was measured by using the Courtenay soft agar colony assay. The four melanomas formed spherical, densely packed spheroids. The volumetric growth rate as well as the plating efficiency in soft agar usually increased with increasing passage number. The radiosensitivity differed significantly among the melanomas. The survival curves for single cells from disaggregated spheroids in the first passage were always similar to those for single cells isolated directly from the surgical specimens. Two of the melanomas showed a significant contact effect as spheroids whereas the other two did not. The spheroids of two of the melanomas showed lower D0 in the third and the sixth passage than in the first passage, whereas the spheroids of the other two melanomas showed similar survival curves in the first and the third passage. There was no clear relationship between the changes in radiosensitivity and the changes in growth rate or plating efficiency. It is concluded that spheroids in the first passage, but not spheroids in later passages, may have the potential to identify differences in clinical radioresponsiveness among tumours.


Br. J. Cancer (1986) **54**, 569–578

### Growth and radiosensitivity of malignant melanoma multicellular spheroids initiated directly from surgical specimens of tumours in man

E.K. Rofstad

*Institute for Cancer Research and The Norwegian Cancer Society, The Norwegian Radium Hospital, Montebello, 0310 Oslo 3, Norway.*

**Summary** The growth and radiosensitivity of multicellular spheroids initiated directly from disaggregated surgical specimens of four human malignant melanomas were studied. The spheroids were grown in liquid-overlay culture for up to 6 passages. Cell survival following irradiation was measured by using the Courtenay soft agar colony assay. The four melanomas formed spherical, densely packed spheroids. The volumetric growth rate as well as the plating efficiency in soft agar usually increased with increasing passage number. The radiosensitivity differed significantly among the melanomas. The survival curves for single cells from disaggregated spheroids in the first passage were always similar to those for single cells isolated directly from the surgical specimens. Two of the melanomas showed a significant contact effect as spheroids whereas the other two did not. The spheroids of two of the melanomas showed lower $D_0$ in the third and the sixth passage than in the first passage, whereas the spheroids of the other two melanomas showed similar survival curves in the first and the third passage. There was no clear relationship between the changes in radiosensitivity and the changes in growth rate or plating efficiency. It is concluded that spheroids in the first passage, but not spheroids in later passages, may have the potential to identify differences in clinical radioresponsiveness among tumours.

Multicellular spheroids are an *in vitro* tumour model system representing an intermediate level of complexity between monolayer cell cultures and solid tumours (Sutherland *et al.*, 1970; Sutherland & Durand, 1976). The model has several qualities making it interesting in studies of human tumour radiobiology (Steel & Courtenay, 1983). Spheroids initiated from different cell or tumour lines show individual and characteristic growth parameters, e.g. volume-doubling time, cell cycle distribution, cell density and intercellular adhesiveness (Carlsson *et al.*, 1983; Rofstad *et al.*, 1986*a*). Large spheroids have diffusion gradients for oxygen, glucose and other nutrients, resulting in necrotic areas, radiobiologically hypoxic cells and cells at acid pH (Sutherland & Durand, 1973; Acker 1984). Important radiobiological phenomena such as contact effect (Durand & Sutherland, 1972), repair processes (Sutherland & Durand, 1973; Durand & Sutherland, 1976) and reoxygenation (Durand & Sutherland, 1976) have been demonstrated for several spheroid systems. Moreover, the radiation response of spheroids initiated from human tumour xenografts has been shown to be similar to that of the parent tumours (Rofstad *et al.*, 1986*b,c*).

Correspondence: E.K. Rofstad.
Received 14th April 1986; and in revised form, 27th May 1986.

Generally, spheroids have been initiated from animal or human cell lines established as monolayer cultures (Sutherland & Durand, 1976; Carlsson *et al.*, 1983) or from disaggregated human tumour xenografts (Jones *et al.*, 1982; Twentyman, 1983; West *et al.*, 1984; Rofstad *et al.*, 1986*a*). Recently, there has been some interest in growing spheroids directly from human tumour surgical specimens (Darling *et al.*, 1983; Wibe *et al.*, 1984). Although these studies have shown that the spheroids may maintain several biological characteristics of the parent tumours, little attention has been devoted to studies of the radiosensitivity of such spheroids. The radiosensitivity of spheroids initiated from surgical specimens of four different melanoma patients, measured by using the Courtenay soft agar colony assay (Courtenay & Mills, 1978), is reported in the present communication. There was a dual purpose of the work: (a) to compare the cell survival curves for the spheroids with those for single cells from the same tumours; and (b) to study possible changes in volumetric growth rate and cellular radiosensitivity during serial subculture of the spheroids.

### Materials and methods

### *Tumour tissue*

Tumour tissue from melanoma patients admitted to

© The Macmillan Press Ltd., 1986

**Table I** Malignant melanomas.

| | *Patient* | | *Tumour* | |
|---|---|---|---|---|
| *Melanoma*[a] | *Age (yrs)* | *Sex* | *Form* | *Site* |
| A.M. | 53 | M | Node metastasis | Inguen |
| B.K. | 51 | F | Node metastasis | Inguen |
| F.P. | 32 | F | Node metastasis | Axilla |
| T.D. | 40 | M | Node metastasis | Inguen |

[a]The letters do not refer to the initials of the patients from whom the melanomas were derived.

The Norwegian Radium Hospital was used (Table I). The Department of Surgery was routinely supplying our laboratory with melanoma surgical specimens. The specimens were put into culture medium (4°C) immediately after surgery and then brought to the laboratory. Normal tissue and necrotic areas were removed with scalpels. Tumour fragments were suspended in 20 ml culture medium in a plastic bag and treated for 30 sec with a stomacher ('Lab-Blender 80', Seward Laboratory, London, U.K.) for further mechanical disaggregation. The suspensions were filtered through 45 $\mu$m nylon mesh before centrifugation and resuspension in culture medium. The cell concentration was determined by using a microscope with phase-contrast optics. Cells having an intact and smooth outline with a bright halo were counted as viable. The cell suspensions were divided into several samples of appropriate size. Some samples were used in experiments immediately after a cell suspension was prepared, whereas others were frozen in liquid nitrogen and stored. The four specimens used in the present work all yielded suspensions with $1 \times 10^8$–$1 \times 10^9$ cells. They were especially selected for this study because preliminary investigations had shown that (a) the cells formed densely packed, spherical spheroids that grew at an acceptable rate in liquid-overlay culture; and (b) the plating efficiency of the cells in soft agar was higher than 5%.

*Spheroids*

The spheroids were grown in Ham's F12 culture medium supplemented with 20% foetal calf serum, 250 mg l$^{-1}$ penicillin and 50 mg l$^{-1}$ streptomycin (Gibco-Biocult, Glasgow, Scotland). Care was taken to ensure that the experimental procedure and the growth conditions were equal for all spheroid cultures. Approximately $1.5 \times 10^6$ cells in 30 ml medium were seeded in 75 cm$^2$ plastic tissue culture flasks (Falcon, Oxnard, USA) coated with a thin layer (3 ml per flask) of 1% agar (Bacto Agar, Difco, Detroit, USA). The flasks were then agitated (10 periods per min) at 37°C for 2 hours using a tilting platform (Rotary Mixer, Cenco. Inst., Breda, The Netherlands), and aggregates, approximately 50 $\mu$m in diameter, were formed. One day later, about 200 aggregates were transferred to 75 cm$^2$ coated culture flasks and cultivated in 30 ml medium at an atmosphere of 5% $O_2$, 5% $CO_2$ and 90% $N_2$ (liquid-overlay culture). The agar coating prevented attachment of spheroids to the bottom of the flasks. The culture medium was changed three times a week. The diameters of the spheroids were measured by using an ocular micrometer in an inverted phase contrast microscope. Growth curves were based on measurements of 40 spheroids chosen at random at each point of time. The spheroids had a diameter of $140 \pm 10\,\mu$m when they were irradiated. The number of cells per spheroid was measured to be in the range 800–1,200. Spheroids of this size had not developed central necrosis, as ascertained from examination of histological sections stained with eosin and haematoxylin according to standard procedures.

*Irradiation*

A Siemens 'Stabilipan' X-ray unit, operated at 220 kV, 19–20 mA, and with 0.5 mm Cu filtration, was used for irradiation. Spheroid and cell suspensions were irradiated under aerobic conditions at a dose rate of 3.0 Gy min$^{-1}$. The suspensions were kept in glass Carrel flasks during exposure. The irradiation was performed at room temperature.

*Colony assay*

Cell survival was measured by using the soft agar colony assay developed by Courtenay & Mills (1978). Single cells were obtained by treating the spheroids with a 0.05% trypsin/0.02% EDTA solution for 10 min at 37°C. The spheroids were disaggregated completely by this treatment and almost 100% of the cells appeared to be morphologically intact. The soft agar was prepared from powdered agar and culture medium with 20% foetal calf serum, 250 mg l$^{-1}$ penicillin and 50 mg l$^{-1}$ streptomycin. Erythrocytes from August rats and melanoma cells were added as previously described (Rofstad, 1981). Aliquots of 1 ml of soft agar with the appropriate number of melanoma cells were seeded in plastic tubes (Falcon 2057 tubes, Oxnard, USA). The cells were incubated at 37°C for 4–5 weeks in an atmosphere of 5% $O_2$, 5% $CO_2$ and 90% $N_2$. Culture medium (2 ml) was added on the top of the agar 5 days after seeding and then changed weekly. Colonies >50 cells were counted by using a stereomicroscope. Care was taken to avoid potential pitfalls that may occur in the Courtenay assay when used to study survival of cells from human tumour surgical specimens. These pitfalls have been discussed in detail previously (Rofstad *et al.*, 1985*a*). Survival curves and parameters ($D_0$, $n$) were determined by using the multitarget-single-hit model.

*Nomenclature and experimental design*

The melanomas were given the initials A.M., B.K., F.P. and T.D. (These letters do not refer to the initials of the patients from whom the melanomas were derived). The term p0 refers to single cells derived directly from the surgical specimens; the terms p1, p3 and p6 to cells grown as spheroids for 1, 3 and 6 passages, respectively.

The radiosensitivity of p0 cells was studied by using cells from newly prepared suspensions as well as cells stored in liquid nitrogen. Cells stored in liquid nitrogen were always used to initiate p1 spheroids and thereafter the cells were propagated as spheroids for up to six passages for growth and radiosensitivity studies. Two series of spheroid cultures, initiated from different frozen samples and propagated independently, were studied for each melanoma.

### Results

Growth curves for the spheroids are shown in Figure 1. The growth was exponential up to a volume of $1 \times 10^7 - 2 \times 10^7\,\mu m^3$ (diameter of 270–340 $\mu$m) and then the growth rate levelled off. The volume-doubling times during the exponential growth phase were within the range 2–8 days. The growth rate was higher for serially passaged than for p1 spheroids for three of the melanomas (A.M., B.K., T.D.), whereas the fourth (F.P.) showed no increase in the growth rate with increasing passage number. Apart from obvious differences relating to vascular properties, the histological and cytological appearance of the spheroids was remarkably similar to that of the parent tumours. The plating efficiency in soft agar was usually higher for cells from spheroids than for cells derived directly from the surgical specimens and increased somewhat with increasing spheroid passage number (Table II).

X-ray survival curves for cells derived directly from the surgical specimens are presented in Figure 2. Experiments with stored cells gave similar results as experiments with fresh cells. The radiosensitivity varied significantly among the different melanomas. $D_0$ ranged from 0.80 to 1.15 Gy and the extrapolation number from 1.6 to 8.3 (Table III).

Figures 3–6 show X-ray survival curves for the melanomas grown as spheroids. The spheroids were disaggregated either immediately before or immediately after irradiation. Cells from p1 spheroids irradiated immediately after disaggregation always showed survival curves similar to those for the corresponding p0 cells. The survival curves for spheroids disaggregated immediately before and immediately after irradiation were not significantly different for the A.M. and B.K. melanomas, whereas the F.P. and T.D. melanomas showed a significant contact effect. The A.M. and F.P. melanomas showed similar survival curves in p1 and p3, whereas the B.K. and T.D. melanomas showed lower $D_0$ in p3 and p6 than in p1. The survival curves for p3 and p6 spheroids were not significantly different. The changes in the survival curves with increasing passage number were similar for the two independent series of spheroid cultures for each melanoma. A contact effect did not appear or disappear during serial spheroid growth. There was no evidence of presence of hypoxic cells in the spheroids. The survival curve parameters are presented in Table III.

**Table II** Plating efficiencies.[a]

| | *PE* (%) | | | |
|---|---|---|---|---|
| *Melanoma* | *p0* | *p1* | *p3* | *p6* |
| A.M. | 5.4–8.5 | 10.1–17.6 | 25.1–30.3 | 24.1–31.6 |
| B.K. | 15.9–21.3 | 17.8–25.0 | 12.5–22.7 | 18.8–23.1 |
| F.P. | 8.5–12.1 | 10.9–16.2 | 20.0–26.3 | 21.0–24.8 |
| T.D. | 6.2–9.2 | 6.5–12.0 | 8.9–13.1 | 11.1–14.0 |

[a]Ranges.

**Figure 1** Growth curves for human melanoma multicellular spheroids. Each point is based on 40 spheroids. Vertical bars indicate s.e.

**Table III** Survival curve parameters.[a]

| | *Cells* | | *Spheroids* | |
|---|---|---|---|---|
| *Tumour* | $D_0$ *(Gy)* | *n* | $D_0$ *(Gy)* | *n* |
| A.M. p0 | 0.94±0.05 | 2.2±0.6 | | |
| A.M. p1 | 0.91±0.03 | 2.6±0.4 | 0.94±0.06 | 2.4±0.7 |
| A.M. p3 | 0.92±0.05 | 2.5±0.8 | 1.01±0.06 | 1.7±0.6 |
| B.K. p0 | 0.99±0.06 | 8.3±2.7 | | |
| B.K. p1 | 1.01±0.08 | 8.0±3.9 | 0.97±0.08 | 8.2±4.4 |
| B.K. p3 | 0.69±0.07 | 11.7±9.6 | 0.86±0.07 | 3.3±1.6 |
| B.K. p6 | 0.78±0.14 | 5.1±6.9 | 0.76±0.06 | 6.5±3.3 |
| F.P. p0 | 0.80±0.03 | 1.8±0.4 | | |
| F.P. p1 | 0.77±0.06 | 2.3±0.9 | 1.05±0.07 | 2.9±1.2 |
| F.P. p3 | 0.78±0.05 | 2.3±0.8 | 1.01±0.08 | 3.7±1.8 |
| T.D. p0 | 1.15±0.05 | 1.6±0.3 | | |
| T.D. p1 | 1.15±0.06 | 1.6±0.4 | 1.30±0.08 | 2.3±0.7 |
| T.D. p3 | 0.87±0.06 | 1.3±0.4 | 0.87±0.08 | 3.6±2.2 |
| T.D. p6 | 0.82±0.04 | 1.9±0.5 | 0.89±0.07 | 3.3±1.7 |

[a]Mean values±s.e. Survival curves were fitted to the data by using the multitarget-single-hit model.

**Figure 2** X-ray survival curves for cells from human melanomas. Results from two independent experiments are presented for each melanoma. The closed and open symbols refer to cells from newly prepared suspensions and cells stored in liquid nitrogen, respectively. Each survival level was calculated from the mean number of colonies in four tubes with irradiated and four tubes with unirradiated cells.

**Figure 3** X-ray survival curves for multicellular spheroids of the A.M. human melanoma. The spheroids were disaggregated immediately before (●, ○) or immediately after (■, □) irradiation. Results from two independent experiments of each category are presented. The closed and open symbols refer to spheroid series originally initiated from two different frozen cell samples. Each survival level was calculated from the mean number of colonies in four tubes with irradiated and four tubes with unirradiated cells. The dashed curves are the survival curve for A.M. p0 redrawn from **Figure 2.**

**Figure 4** X-ray survival curves for multicellular spheroids of the B.K. human melanoma. The spheroids were disaggregated immediately before (●, ○) or immediately after (■, □) irradiation. Results from two independent experiments of each category are presented. The closed and open symbols refer to spheroid series originally initiated from two different frozen cell samples. Each survival level was calculated from the mean number of colonies in four tubes with irradiated and four tubes with unirradiated cells. The dashed curves are the survival curve for B.K. p0 redrawn from **Figure 2**.

**Figure 5** X-ray survival curves for multicellular spheroids of the F.P. human melanoma. The spheroids were disaggregated immediately before (●, ○) or immediately after (■, □) irradiation. Results from two independent experiments of each category are presented. The closed and open symbols refer to spheroid series originally initiated from two different frozen cell samples. Each survival level was calculated from the mean number of colonies in four tubes with irradiated and four tubes with unirradiated cells. The dashed curves are the survival curve for F.P. p0 redrawn from **Figure 2**.

**Figure 6** X-ray survival curves for multicellular spheroids of the T.D. human melanoma. The spheroids were disaggregated immediately before (●, ○) or immediately after (■, □) irradiation. Results from two independent experiments of each category are presented. The closed and open symbols refer to spheroid series originally initiated from two different frozen cell samples. Each survival level was calculated from the mean number of colonies in four tubes with irradiated and four tubes with unirradiated cells. The dashed curves are the survival curve for T.D. p0 redrawn from **Figure 2**.

### Discussion

The four melanomas formed densely packed, spherical multicellular spheroids that grew exponentially up to a volume of $1\times10^7$–$2\times10^7\,\mu m^3$ in liquid-overlay culture. The morphology of the spheroids was similar to that illustrated previously for spheroids from the E.E. human melanoma xenograft (Rofstad *et al.*, 1985*b*). Histologically the spheroids were similar to the parent tumours in the donor patients. Both the spheroids and the parent tumours stained positive for melanin. The spheroids had attained a diameter of $140\pm10\,\mu m$ at irradiation. Spheroids of this size had not developed central necrosis. Evidence that the spheroids contained radiobiologically hypoxic cells was not found. Consequently, the spheroids were models of the aerobic compartments of the parent tumours.

The plating efficiency in soft agar was usually higher for cells from disaggregated spheroids than for p0 cells. Moreover, the plating efficiency as well as the volumetric growth rate tended to increase with increasing number of passages of the spheroids. Yuhas & Li (1978) have studied the growth in liquid-overlay culture of spheroids initiated from seven murine solid tumours and concluded that the growth fraction was the major determinant of the volumetric growth rate. Previous studies in our laboratory of spheroids initiated from human melanoma xenografts have also shown that differences in volume-doubling time among different spheroid cultures are mainly a consequence of different growth fractions (Rofstad *et al.*, 1986*a*). One possible explanation of the present observations may therefore be that the culture conditions *in vitro* through adequate nutrients stimulated cell proliferation, whereby the growth fraction as well as the fraction of clonogenic cells increased. However, there is evidence from studies of human melanoma xenografts that distinctly different stem-cell subpopulations may be predominant in tumours and in the corresponding spheroids (Rofstad *et al.*, 1986*a*). Consequently, it cannot be excluded that the changes in plating efficiency and volumetric growth rate observed here were due to some stem-cell subpopulations being favoured by the growth conditions *in vitro*.

The Courtenay soft agar colony assay was used to measure cell survival after irradiation. Previous work has revealed some pitfalls in the assay when used to study survival of cells derived directly from human tumour surgical specimens (Rofstad *et al.*, 1985*a*). However, when the necessary precautions are taken to avoid the pitfalls, the assay gives reliable survival curves (Rofstad *et al.*, 1985*a*). Thus, the shape of the survival curves established in the present work was similar to that of survival curves reported for melanoma cells from established lines and xenografts (Rofstad, 1986). The reproducibility of the assay was adequate as indicated by the coinciding results in independent experiments performed with cells from the same melanoma. Moreover, the four melanomas showed individual and characteristic survival curves varying significantly in $D_0$ and $n$. These observations suggest that differences in radiosensitivity among cell populations and spheroid cultures derived directly from melanoma surgical specimens can be identified by using the Courtenay colony assay.

Another important question is to what extent the radiosensitivity of such spheroid cultures mirrors the radioresponsiveness *in vivo* of the parent melanomas. Melanomas have often been classified as radioresistant (Fertil & Malaise, 1981; Deacon *et al.*, 1984), but the radioresistance of melanomas has been questioned by several clinicians (Lobo *et al.*, 1981; Trott *et al.*, 1981). Recent reviews conclude that melanomas constitute a heterogeneous tumour group with very variable clinical radioresponsiveness (Habermalz, 1981; Harwood & Cummings, 1981; Rofstad, 1986). The survival curve parameters in Table III are therefore within the expected range for melanomas. The present donor patients were not subjected to radiotherapy, and hence a direct comparison with the clinical radioresponsiveness is not possible. However, previous work has indicated that the radiosensitivity of spheroids initiated directly from human melanoma xenografts reflects the radioresponsiveness *in vivo* of the parent tumours (Rofstad *et al.*, 1986*c*). Moreover, melanoma xenografts that showed a contact effect *in vivo* also showed a contact effect as spheroids and *vice versa*, i.e. there was complete agreement between the spheroid and the tumour experiments (Rofstad *et al.*, 1986*c*). Two of the melanomas studied here showed a significant contact effect whereas the other two did not, suggesting that spheroids initiated from surgical specimens also have the potential to identify a possible contact effect. Furthermore, the survival curves for cells from disaggregated p1 spheroids were always similar to those for the corresponding p0 cells, indicating that spheroid growth for one passage did not alter the radiosensitivity of the cells significantly. Consequently, the results from the present work and our xenograft work (Rofstad *et al.*, 1986*c*) give some evidence that the radiosensitivity of p1 spheroid cultures initiated from melanoma surgical specimens may reflect the radioresponsiveness *in vivo* of the parent tumours.

The $D_0$ was lower for p3 and p6 spheroids than for p1 spheroids for two of the melanomas, whereas the spheroids of the other two melanomas showed similar $D_0$ in p1 and p3. The two independent spheroid series for each melanoma showed the same changes in radiosensitivity with increasing passage number, suggesting that the changes were not a result of random biological events, but were rather governed by the culture conditions *in vitro*. However, there was no clear relationship between the changes in radiosensitivity and the changes in volumetric growth rate or plating efficiency. This indicates that the changes in radiosensitivity probably were not just a secondary effect of changes in the cell proliferation. In any case, the present work shows that at least some spheroid cultures grown for more than one passage *in vitro* probably do not mirror the radioresponsiveness *in vivo* of the parent melanomas.

One objective of the present work was to discuss whether spheroid cultures initiated from surgical specimens may be used beneficially to predict the clinical radioresponsiveness of tumours. There is some evidence that the clinical radioresponsiveness of tumours is related to the initial slope of the cell survival curve (Fertil & Malaise, 1981; 1985; Deacon *et al.*, 1984). If this is so, the clinical radioresponsiveness may be predicted more accurately from p1 spheroids than from p0 single cells. Spheroids in p1 show the same cellular radiosensitivity as p0 cells and, in addition, have the potential to identify and record a possible contact effect. However, our general experience, which is in agreement with recently published studies (Jones *et al.*, 1982; Wibe *et al.*, 1984), is that most surgical specimens under the present growth conditions do not give rise to a sufficient number of p1 spheroids for radiosensitivity testing. This problem cannot be overcome by reculturing the spheroids for a sufficient number of passages since the cellular radiosensitivity may change significantly during serial growth. Nevertheless, our data are encouraging and should stimulate research aimed at finding factors that will increase spheroid formation and growth *in vitro*.

Financial support from The Norwegian Cancer Society, The Norwegian Research Council for Science and the Humanities, and The Nansen Scientific Fund is gratefully acknowledged.

### References

ACKER, H. (1984). Microenvironmental conditions in multicellular spheroids grown under liquid-overlay tissue culture conditions. In *Spheroids in Cancer Research. Methods and Perspectives*, Acker, H. & 3 others (eds) p. 116. Springer Verlag: Berlin and Heidelberg.

CARLSSON, J., NILSSON, K., WESTERMARK, B. & 7 others (1983). Formation and growth of multicellular spheroids of human origin. *Int. J. Cancer*, **31**, 523.

COURTENAY, V.D. & MILLS, J. (1978). An *in vitro* colony assay for human tumours grown in immune-suppressed mice and treated *in vivo* with cytotoxic agents. *Br. J. Cancer*, **37**, 261.

DARLING, J.L., OKTAR, N. & THOMAS, D.G.T. (1983). Multicellular tumour spheroids derived from human brain tumours. *Cell Biol. Int. Rep.*, **7**, 23.

DEACON, J., PECKHAM, M.J. & STEEL, G.G. (1984). The radioresponsiveness of human tumours and the initial slope of the cell survival curve. *Radiother. Oncol.*, **2**, 317.

DURAND, R.E. & SUTHERLAND, R.M. (1972). Effects of intercellular contact on repair of radiation damage. *Exp. Cell Res.*, **71**, 75.

DURAND, R.E. & SUTHERLAND, R.M. (1976). Repair and reoxygenation following irradiation of an *in vitro* tumor model. *Int. J. Radiat. Oncol. Biol. Phys.*, **1**, 1119.

FERTIL, B. & MALAISE, E.P. (1981). Inherent cellular radiosensitivity as a basic concept for human tumor radiotherapy. *Int. J. Radiat. Oncol. Biol. Phys.*, **7**, 621.

FERTIL, B. & MALAISE, E.P. (1985). Intrinsic radiosensitivity of human cell lines is correlated with radioresponsiveness of human tumors: Analysis of 101 published survival curves. *Int. J. Radiat. Oncol. Biol. Phys.*, **11**, 1699.

HABERMALZ, H.J. (1981). Irradiation of malignant melanoma: Experience in the past and present. *Int. J. Radiat. Oncol. Biol. Phys.*, **7**, 131.

HARWOOD, A.R. & CUMMINGS, B.J. (1981). Radiotherapy for malignant melanoma: A re-appraisal. *Cancer Treat. Rev.*, **8**, 271.

JONES, A.C., STRATFORD, I.J., WILSON, P.A. & PECKHAM, M.J. (1982). *In vitro* cytotoxic drug sensitivity testing of human tumour xenografts grown as multicellular tumour spheroids. *Br. J. Cancer*, **46**, 870.

LOBO, P.A., LIEBNER, E.J., CHAO, J.H. & KANJI, A.M. (1981). Value of radiotherapy in the management of malignant melanoma. *Int. J. Radiat. Oncol. Biol. Phys.*, **7**, 21.

ROFSTAD, E.K. (1981). Radiation response of the cells of a human malignant melanoma xenograft. Effect of hypoxic cell radiosensitizers. *Radiat. Res.*, **87**, 670.

ROFSTAD, E.K. (1986). Radiation biology of malignant melanoma. *Acta Radiol. Oncol.*, **25**, 1.

ROFSTAD, E.K., WAHL, A. & BRUSTAD, T. (1985*b*). Heat response of human melanoma multicellular spheroids: Comparisons with single cells and xenografted tumors. *Radiat. Res.*, **102**, 324.

ROFSTAD, E.K., WAHL, A. & BRUSTAD, T. (1986*b*). Radiation response of human melanoma multicellular spheroids measured as single cell survival, growth delay, and spheroid cure: Comparisons with the parent tumor xenograft. *Int. J. Radiat. Oncol. Biol. Phys.*, (in press).

ROFSTAD, E.K., WAHL, A. & BRUSTAD, T. (1986*c*). Radiation response of multicellular spheroids initiated from five human melanoma xenograft lines. Relationship to the radioresponsiveness *in vivo*. *Br. J. Radiol.*, (in press).

ROFSTAD, E.K., WAHL, A., DAVIES, C. DE L. & BRUSTAD, T. (1986*a*). Growth characteristics of human melanoma multicellular spheroids in liquid-overlay culture: Comparisons with the parent tumour xenografts. *Cell Tissue Kinet.*, **19**, 205.

ROFSTAD, E.K., WAHL, A., TVEIT, K.M., MONGE, O.R. & BRUSTAD, T. (1985*a*). Survival curves after X-ray and heat treatments for melanoma cells derived directly from surgical specimens of tumours in man. *Radiother. Oncol.*, **4**, 33.

STEEL, G.G. & COURTENAY, V.D. (1983). The radiobiology of human tumour cells. In *The Biological Basis of Radiotherapy*, G.G. Steel & 2 others (eds) p. 123. Elsevier Science Publishers B.V.: Amsterdam.

SUTHERLAND, R.M. & DURAND, R.E. (1973). Hypoxic cells in an *in vitro* tumour model. *Int. J. Radiat. Biol.*, **23**, 235.

SUTHERLAND, R.M. & DURAND, R.E. (1976). Radiation response of multicell spheroids – an *in vitro* tumour model. *Curr. Top. Rad. Res. Quart.*, **11**, 87.

SUTHERLAND, R.M., INCH, W.R., MCCREDIE, J.A. & KRUUV, J. (1970). A multi-component radiation survival curve using an *in vitro* tumour model. *Int. J. Radiat. Biol.*, **18**, 491.

TROTT, K.R., VON LIEVEN, H., KUMMERMEHR, J., SKOPAL, D., LUKACS, S., BRAUN-FALCO, O. & KELLERER, A.M. (1981). The radiosensitivity of malignant melanomas. II. Clinical studies. *Int. J. Radiat. Oncol. Biol. Phys.*, **7**, 15.

TWENTYMAN, P.R. (1983). Exclusion of host cells during spheroid formation from disaggregated solid tumours. *Br. J. Cancer*, **47**, 541.

WEST, C.M.L., SANDHU, R.R. & STRATFORD, I.J. (1984). The radiation response of V79 and human tumour multicellular spheroids – cell survival and growth delay studies. *Br. J. Cancer*, **50**, 143.

WIBE, E., BERG, J.P., TVEIT, K.M., NESLAND, J.M. & LUNDE, S. (1984). Multicellular spheroids grown directly from human tumour material. *Int. J. Cancer*, **34**, 21.

YUHAS, J.M. & LI, A.P. (1978). Growth fraction as the major determinant of multicellular tumor spheroid growth rates. *Cancer Res.*, **38**, 1528.